# Transcriptome profiling of brain myeloid cells revealed activation of *Itgal*, *Trem1*, and *Spp1* in western diet-induced obesity

**DOI:** 10.1186/s12974-019-1527-z

**Published:** 2019-08-19

**Authors:** Hongtian Yang, Leah C. Graham, Alaina M. Reagan, Weronika A. Grabowska, William H. Schott, Gareth R. Howell

**Affiliations:** 1 The Jackson Laboratory, Bar Harbor, ME USA; 2 Sackler School of Graduate Biomedical Sciences, Tufts University School of Medicine, Boston, MA USA; 3 Graduate School of Biomedical Sciences and Engineering, University of Maine, Orono, ME USA

**Keywords:** Obesity, Western diet, Myeloid cells, Microglia, Monocytes, Neutrophils, *Spp1*, Osteopontin

## Abstract

**Background:**

Environmental factors are critical in the development of age-related cognitive decline and dementia. A western diet (WD) can cause nutrient deficiency and inflammation that could impact cognition directly. It is increasingly recognized that innate immune responses by brain myeloid cells, such as resident microglia, and infiltrating peripheral monocytes/macrophages may represent an essential link between a WD, cognitive decline, and dementia. Our previous data demonstrated that chronic consumption of a WD induced inflammation through brain myeloid cells in aging mice and a mouse model of Alzheimer’s disease (AD). However, the subtypes of myeloid cells that contribute to the WD-induced inflammation remain unclear.

**Methods:**

C57BL/6J (B6), myeloid cell reporter mice (B6.*Ccr2*^*RFP/+*^*Cx3cr1*^*GFP/+*^), and *Ccr2*-deficient mice (B6.*Ccr2*^*RFP/RFP*^) were fed a WD or a control chow diet (CD) from 2 to 6 or 12 months of age. CD11b+CD45^lo^ and CD11b+CD45^hi^ cells from WD- and CD-fed B6 or *Ccr2*-deficient mice were characterized using flow cytometry, RNA-sequencing, and immunofluorescence.

**Results:**

*Ccr2*::RFP expressing myeloid cells were significantly increased in brains of WD- compared to CD-fed mice, but were not elevated in *Ccr2*-deficient WD-fed mice. The percent of CD11b+CD45^hi^ cells was significantly increased in WD- compared to CD-fed mice. Comparison of RNA-sequencing data with immune cell data in ImmGen supports that CD11b+CD45^hi^ cells from WD-fed mice are enriched for peripheral monocytes and neutrophils. Ingenuity pathway analysis predicted these cells elicit proinflammatory responses that may be damaging to the brain. Using stringent criteria for gene expression levels between CD11b+CD45^hi^ and CD11b+CD45^lo^ cells, we identified approximately 70 genes that we predict are uniquely expressed in infiltrating cells, including *Itgal*, *Trem1*, *and Spp1* (osteopontin, OPN*)*. Finally, we show a significantly greater number of OPN+IBA1– cells in WD- compared to CD-fed mice that we propose are activated neutrophils based on ImmGen data. OPN+IBA1– cells are not significantly increased in *Ccr2*-deficient WD-fed mice.

**Conclusions:**

These data further support the model that peripheral myeloid cells enter the brain in response to diet-induced obesity. Elucidating their contribution to age-related cognitive decline and age-related neurodegenerative diseases should offer new avenues for therapeutic intervention in Alzheimer’s disease and related dementias, where diet/obesity are major risk factors.

**Electronic supplementary material:**

The online version of this article (10.1186/s12974-019-1527-z) contains supplementary material, which is available to authorized users.

## Introduction

Obesity continues to be a major health issue in the USA, with the past decade demonstrating a significantly increasing trend nationwide. From 2015 to 2016, the prevalence of obesity was 39.8% in adults and 18.5% in youths [1]. Estimates for obesity-related healthcare costs range from $147 billion to nearly $210 billion per year [2, 3]. Genetic predisposition as well as lifestyle choices such as chronic consumption of a western diet and lack of exercise can lead to the development of obesity. A cellular hallmark of obesity is inflammatory circulating macrophages and other immune cells infiltrating into adipose tissue, inducing low-grade inflammation that affects peripheral tissues [4]. Obesity associated-inflammation results in a high incidence of comorbidities, including type 2 diabetes and cardiovascular disease [4].

Obesity is also associated with an increased incidence of cognitive decline and dementia. Studies suggest obesity, particularly mid-life obesity, increases the chances of cognitive decline and Alzheimer’s disease by six-fold [5, 6]. Like inflammation in peripheral tissue, obesity promotes inflammatory responses in the brain, which can then result in progressive cognitive decline [7, 8]. Recent data suggest that innate immune responses mediated by myeloid cells (including brain-resident microglia and infiltrating macrophages from the periphery) likely represent an important link between obesity, cognitive decline, and AD [9–13]. Our previous study showed robust glial responses following chronic consumption of a western diet, including an increase in IBA1+TREM2+ myeloid cells in the brains of aging C57BL/6J (B6) and B6.*APP/PS1* mice [10]. Additional studies in mouse models have shown that a high-fat diet is associated with neuroinflammation by both microglia [9, 14–16] and infiltrating myeloid cells in the brain [17]. However, it is not clear whether the activity of microglia or infiltrating myeloid cells is beneficial or detrimental during obesity, in part because specifically distinguishing and targeting these myeloid cell subtypes are challenging [18]. Deep characterization of myeloid cell subpopulations (e.g., microglia versus peripheral monocytes) in the context of obesity would help define the different cell types to test their beneficial or damaging functions. Understanding specific cell parameters under different conditions will allow targeted therapeutic interventions for obesity and related neurological diseases that share similar neuroinflammatory components.

In this study, we provide data to support peripheral myeloid cell infiltration into the brain during chronic western diet consumption in a CCR2-dependent manner. Traditionally, myeloid cells in the brain have been characterized by the presence of CD11b and differential levels of CD45—with CD45^hi^ cells representing peripheral myeloid cells and CD45^lo^ cells representing microglia. Therefore, we characterized the transcriptomes of CD11b+CD45^lo^ and CD11b+CD45^hi^ cells using flow cytometry and RNA sequencing. Analyses of flow cytometry and transcriptomic data support our model that CD11b+CD45^hi^ cells from WD-fed mice are enriched for multiple subtypes of responding microglia and infiltrating immune cells including monocytes/macrophages and neutrophils. We identified a set of 73 genes that are highly enriched in CD11b+CD45^hi^ compared to CD11b+CD45^lo^ cells that provide a new resource for more precisely defining the roles of infiltrating myeloid cells in brain health and disease.

## Methods

### Animals

All methods are in accordance with The Jackson Laboratory Institutional Animal Care and Use Committee (IACUC) approved protocols. All the mice used in this study including C57BL/6J (B6, stock no. 000664), *Ccr2*^*RFP/RFP*^*Cx3cr1*^*GFP/GFP*^ (stock no. 032127), *Ccr2*^*RFP/RFP*^ (stock no. 017586), and B6.*APP/PS1* (stock no. 034832) were obtained from The Jackson Laboratory. All mouse strains were maintained on a B6 genetic background. Homozygous *Ccr2*^*RFP/RFP*^*Cx3cr1*^*GFP/GFP*^ mice were crossed to B6 to make heterozygous *Ccr2*^*RFP/+*^*Cx3cr1*^*GFP/+*^ mice for visualization of RFP+ and GFP+ cells. Homozygous *Ccr2*^*RFP/RFP*^ mice were crossed to B6 to generate heterozygous *Ccr2*^*RFP/+*^ mice, which were then intercrossed to generate *Ccr2*^*RFP/RFP*^ referred to as (*Ccr2*-KO) mice and *Ccr2*^*+/+*^ referred to as wild type (WT) littermate controls. Both males and females were used in all histological experiments, but only males were used for flow cytometry and fluorescent activated cell sorting (FACS). All mice were maintained on a 12/12 h light/dark cycle. All aged (20 months), young (3 months), and middle-aged (12 months) chow diet (CD) cohorts were maintained from wean on standard LabDiet® 5 K52 (control chow diet, CD) [10]. The 12-month western diet (WD) cohort was switched to TestDiet® 5 W80 diet adapted from TestDiet® 5TLN with added high fructose corn syrup, lower fiber, and increased milk protein and fat at 2 months [10]. Daily monitoring of mice via routine welfare check was carried out to determine their general well-being. Approximately 10% of WD-fed mice developed dermatitis and were eliminated from this study using an IACUC-approved CO_2_ euthanasia protocol.

### Mouse perfusion and tissue preparation

Tissues from all WD studies were collected at 3 months and 12 months of age. Tissue from B6.*APP/PS1* and aged B6 mice were collected at 6 months and 20 months, respectively. Mice were anesthetized with a lethal dose of ketamine/xylazine, transcardially perfused with 1X phosphate-buffered saline (PBS), and brains carefully dissected and hemisected at the midsagittal plane. One hemisphere was snap-frozen, and the other half immersion fixed in 4% paraformaldehyde (PFA) for two nights at 4 °C. After fixation, the brains were rinsed in 1X PBS, immersed in 30% sucrose/PBS overnight at 4 °C, frozen in OCT, and cryosectioned coronally at a thickness of 20 μm.

### Immunofluorescence

Sections were dried for 15 min (minutes) at 37 °C followed by one 10-min wash in 1X PBT (1% PBS + 1% Triton 100X) at room temperature and incubated in primary antibodies: goat polyclonal anti-Osteopontin (1:100, Thermofisher Scientific, #PA1-25152), rabbit polyclonal anti-IBA1 (1:250, Wako #019-19741), diluted in 1X PBT + 10% normal goat, or normal donkey serum overnight at 4 °C. After incubation with primary antibodies, all sections were rinsed three times with 1X PBT for 10 min and incubated for 2 h (hours) in the appropriate secondary antibodies (donkey anti-goat Alexa Fluor 568/647, donkey/goat anti-rabbit Alexa Fluor 488, all 1:1000 from Thermo Fisher Scientific). Tissue was then washed three times with 1X PBT for 10–15 min, incubated with DAPI for 5 min and mounted in Poly aquamount (Polysciences). Sections collected from *Ccr2*^*RFP/+*^*Cx3cr1*^*GFP/+*^ brains were simply stained with DAPI, rinsed, and mounted.

### Imaging, image quantification, and statistical analysis

All images were taken using either the Leica SP8 confocal microscope or the Zeiss Axio Imager Z2. All imaging was set up using sections from CD-fed mice as standard. For each labeled protein, all images were captured using identical parameters. Only cells with DAPI staining were included for cell counts. For cell counts in *Cx3cr1*^*GFP/+*^*Ccr2*^*RFP/+*^ mice and quantification of IBA1+ cells in *Ccr2*-KO mice, three to four images within the prefrontal and motor cortex regions were assessed for each mouse (three to four images/mouse), and an average of cell counts per image was determined. Four to six mice were included for each group (*n* = 4–6/group). Increased background was observed in the RFP channel in *Cx3cr1*^*GFP/+*^*Ccr2*^*RFP/+*^ mice, preventing accurate quantification of GFP–RFP+ cells. High background may result from increased autofluorescence in aging mice or mice under chronic consumption of WD. GFP+RFP+ cells were quantified as a measurement of the extent to which peripheral myeloid cells infiltrate into the brain. The GFP+RFP− cell counts were calculated by subtracting the number of GFP+RFP+ cells from that of the total GFP+ cells. Cell counting was performed manually in FIJI by two blinded investigators.

For OPN cell counts, given the reduced number of OPN+ cells compared to GFP+ and RFP+ cells, seven images from the cortical region from the retrosplenial area (ventral part) to temporal associated areas were assessed for each mouse (seven images/mouse), and a sum of cell counts from these images was determined. Three to four mice were included for each group (*n* = 3–4/group). In WT mice fed the WD or CD, OPN signal was visualized by Alexa Fluor 568 and IBA1 Alexa Fluor 488, imaged and presented in the red and green channel, respectively. Although *Ccr2*-KO mice show little to minimal RFP signal in the brain due to impaired CCR2-dependent peripheral myeloid cell infiltration, to avoid possible background RFP signal, we avoided using the red channel to collect images from these brains. Therefore, in *Ccr2*-KO mice, OPN signal was visualized by Alexa Fluor 488 and IBA1 by Alexa Fluor 647, imaged in the green and far-red channel but presented in the red and green channel, respectively. Cell counting was performed manually in FIJI by a single investigator.

Student *T* test was conducted for the cell counts in *Cx3cr1*^*GFP/+*^*Ccr2*^*RFP/+*^ mice. Two-way ANOVA followed by Tukey’s HSD post hoc test was performed in counts of IBA1+ and OPN+IBA1+ cells in *Ccr2*-KO and WT mice. One-way ANOVA followed by Tukey’s HSD post hoc test was performed in OPN+IBA1– counts in 3- or 12-month WT mice. *T* test was performed for OPN+IBA1− counts in 12-month *Ccr2*-KO mice. The significance level was defined as *p* value less than 0.05. All statistical analyses were conducted in *R* (version 3.5.1).

### Brain myeloid cell isolation, fluorescent activated cell sorting (FACS) and flow cytometry

Mice were anesthetized with a lethal dose of ketamine/xylazine, transcardially perfused with Hanks’ Balanced Salt Solution (HBSS) without Ca^2+^ and Mg^2+^ (Gibco, cat#14175-095), and the brains were carefully dissected and hemisected in the midsagittal plane while also removing the olfactory bulb and cerebellum. Brains were kept in HBSS on ice until further processing. Using the Neural Tissue Dissociation Kit (Miltenyi Biotec cat#130-092-628), brains were homogenized into solution in a 60 mm dish on ice before being incubated at 37 °C with on and off pipetting for further homogenization. Samples were washed using RPMI+HEPES (Gibco, cat#61870-036, cat#15630080) before being centrifuged (4 °C) at 450 g for 5 min. Cells were suspended in a percoll solution (Sigma Aldrich, cat#P4937) with 10xHBSS and RPMI + HEPES. 1 mL 10% FBS in RPMI was overlayed onto the percoll cell suspension. Samples were spun at 4 °C at 800 g for 15 min and the centrifuge set to the lowest possible acceleration and deceleration. Supernatant was removed and cells were washed with FACS buffer (PBS with 0.5% BSA) twice. Cells were transferred into FACS tubes and resuspended in 100uL FACS buffer. Cells were then blocked with purified rat anti-mouse CD16/CD32 (BD Biosciences, cat#553142) for 10 min on ice to reduce non-specific antibody binding. Following the block, small amounts of cell suspension were set aside for unstained and single stained controls. Samples were then stained with CD45.2 (used 1:100, BioLegend BV421, #109832), CD11b (used 1:200, BD Pharm BV605, BioLegend #101257), CD3e (used 1:100, APC, BDPharm #553066), CD11c (used 1:100, PE,, BioLegend #117308), Ly6c (used 1:200, FITC, BD Biosciences #553104), and Ly6g (used 1:100, PerCP-cy5.5, BD Biosciences #560602) for 1 h. Cells were washed and resuspended with FACS buffer and placed on ice until cell sorting. FACS and flow cytometry was conducted on a FACSAria II fluorescent cell sorter (BD Biosciences). CD11b+CD45+ cells were sorted into CD11b+CD45^lo^ and CD11b+CD45^hi^ cell subsets based on CD45 levels and were directly collected in RLT Lysis Buffer (Qiagen, cat#79216), snap-frozen, and stored at − 80 °C. Two-way ANOVA followed by Tukey's HSD post hoc test was performed for comparisons between groups.

### RNA extraction, library construction, and sequencing

RNA extraction of CD11b+CD45^lo^ and CD11b+CD45^hi^ cells was performed using TRIzol (Invitrogen, cat#15596026) described in previous publications from our lab [19]. Total RNA was purified from the aqueous layer using the QIAGEN miRNeasy mini extraction kit (QIAGEN) according to the manufacturer’s instructions. RNA quality was assessed with the Bioanalyzer 2100 (Agilent Technologies). Poly(A) selected RNA-seq libraries were generated using the TruSeq RNA Sample preparation kit v2 (Illumina) and quantified using qPCR (Kapa Biosystems). Using Truseq V4 SBS chemistry, all libraries were processed for 75 bp paired-end sequencing on the Illumina NextSeq 500 or HiSeq 4000 platform. Each sample was subjected to a quality control step using NGS QC Toolkit v2.3 for the removal of adapters and trimming low-quality bases (Phred < 30) [20]. Next, we used RSEM v1.2.12 to quantify gene expression using the trimmed reads as input [21]. RSEM internally utilizes Bowtie2 v2.2.0 as its aligner [22] with supplied annotations at default parameters against the C57BL/6J mouse genome (mm10).

### Differential gene expression and pathway analyses

Following alignment and expression quantification, differential gene expression analyses were performed using edgeR 3.20.9 [23]. We applied a filtering step to remove genes with low expression by removing any gene that did not have at least one count per million (cpm) for at least two samples. After filtering, trimmed mean of *M* values (TMM) normalization was applied to remove any potential library size biases. Principle component analysis (PCA) was used to determine the major variables in the datasets. Quasi-likelihood *F* test was applied to determine the differential gene expression across the groups. Significantly differentially expressed (DE) genes were defined with a false discovery rate (FDR) less than 0.05 [i.e., -log_10_(FDR) > 1.3], with an absolute fold change (FC) larger than 1.5 for comparisons between CD11b+CD45^lo^ and CD11b+CD45^hi^ cells or with an absolute FC larger than 1.0 for comparisons between WD and CD in either CD11b+CD45^lo^ and CD11b+CD45^hi^ cells. The lower stringency allows for the detection of any subtle effects of diet on both cell populations. All DE gene analyses and quality control were conducted in *R* (version 3.5.1). All DE genes were uploaded into Ingenuity Pathway Analysis (IPA) software for canonical pathway analysis. The resulting canonical pathways were ranked by Fisher’s exact test *p* value, and the top 15 revealed all had Fisher’s exact test *p* value less than 1 × 10^−4^. The top DE genes enriched in CD11b+CD45^lo^ cells were defined as those with expression levels above 100 cpm and at least two-fold higher compared to CD11b+CD45^hi^ cells. The top DE genes in CD11b+CD45^hi^ cells were defined as those with expression levels above 100 cpm and at least 10-fold higher compared to CD11b+CD45^lo^ cells. The top CD11b+CD45^lo^ and CD11b+CD45^hi^ cell-related genes were compared against mouse immune cell RNA-seq datasets at Immunological Genome Project (ImmGen) [24], My Geneset portal [25]. For plotting individual gene expression from ImmGen datasets, gene expression values were downloaded individually from ImmGen RNA-seq Skyline [26] and were replotted using ggplot2 package in *R*. The top CD11b+CD45^hi^ cell-related genes were also uploaded to David: Functional Annotation tool [27], for gene ontology (GO) term analysis with the background genes set as all detectable genes in RNA-seq dataset. Significant GO term was defined using stringent Benjamini p value (BJ) less than 0.05 [i.e., -log_10_(BJ) > 1.3]. The top CD11b+CD45^hi^ cell-related genes were also imported into IPA for generation of gene interaction network.

## Results

### Chronic western diet consumption increased the number of *Ccr2*::RFP+ cells in the brain

**Fig. 1:**
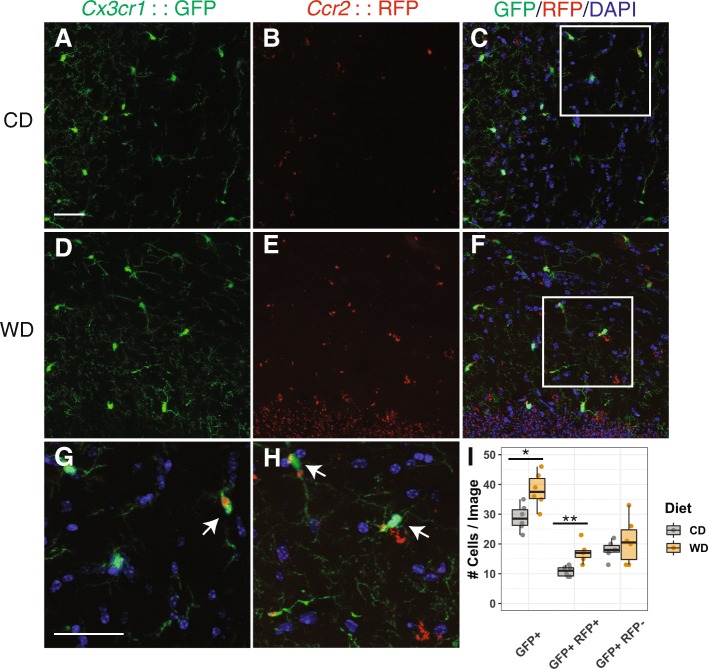
Chronic WD consumption increased *Ccr2*::RFP expressing cells in the brain. **a**–**f** Representative cortical sections from 12-month-old Cx3cr1^GFP/+^Ccr2^RFP/+^ mice fed a CD (**a**–**c**) or WD (**d**–**f**). **g**, **h** Magnified images from boxed region shown on **c** and **f**, respectively. **i** The average number of GFP+, GFP+RFP+ (arrows), and GFP+RFP− cells per image (averaged across at least three images, see the “Methods” section) from mice fed a CD or WD (*T* test was performed in cell counts of each category, *p < 0.05, **p < 0.01). Scale bars, 40 μm

To begin characterizing the myeloid cell populations in obese mice, we employed the *Ccr2*^*RFP/+*^*Cx3cr1*^*GFP/+*^ mouse strain [28]. In this strain, RFP is driven under the *Ccr2* promoter (RFP+) while GFP is driven under the *Cx3cr1* promoter (GFP+). Studies show that GFP+ cells represent the majority of brain myeloid cells particularly resident microglia and some peripheral myeloid cells while RFP+ cells are generally considered peripherally derived [29]. To test the hypothesis that peripheral myeloid cells infiltrate into the brain due to chronic WD consumption, *Ccr2*^*RFP/+*^*Cx3cr1*^*GFP/+*^ mice were fed a western diet (WD) or control chow diet (CD) from 2 to 12 months. Brains from WD- and CD-fed mice were harvested, fixed, sectioned, and GFP+ and RFP+ cells counted. The number of GFP+ cells was significantly greater in WD- compared to CD-fed mice (Fig. 1), suggesting the overall myeloid cell population in the brain increased under WD consumption. Interestingly, the number of GFP+RFP+ cells was also significantly greater in WD- compared to CD-fed mice, suggesting increased infiltration of peripheral myeloid cells into the brain in WD. However, the number of GFP+RFP− cells, likely resident microglia, was not significantly different between WD- and CD-fed mice. These data suggest that a significant increase in brain myeloid cells in response to WD-induced obesity is primarily due to an increase in infiltrating myeloid cells from the periphery.

**Fig. 2:**
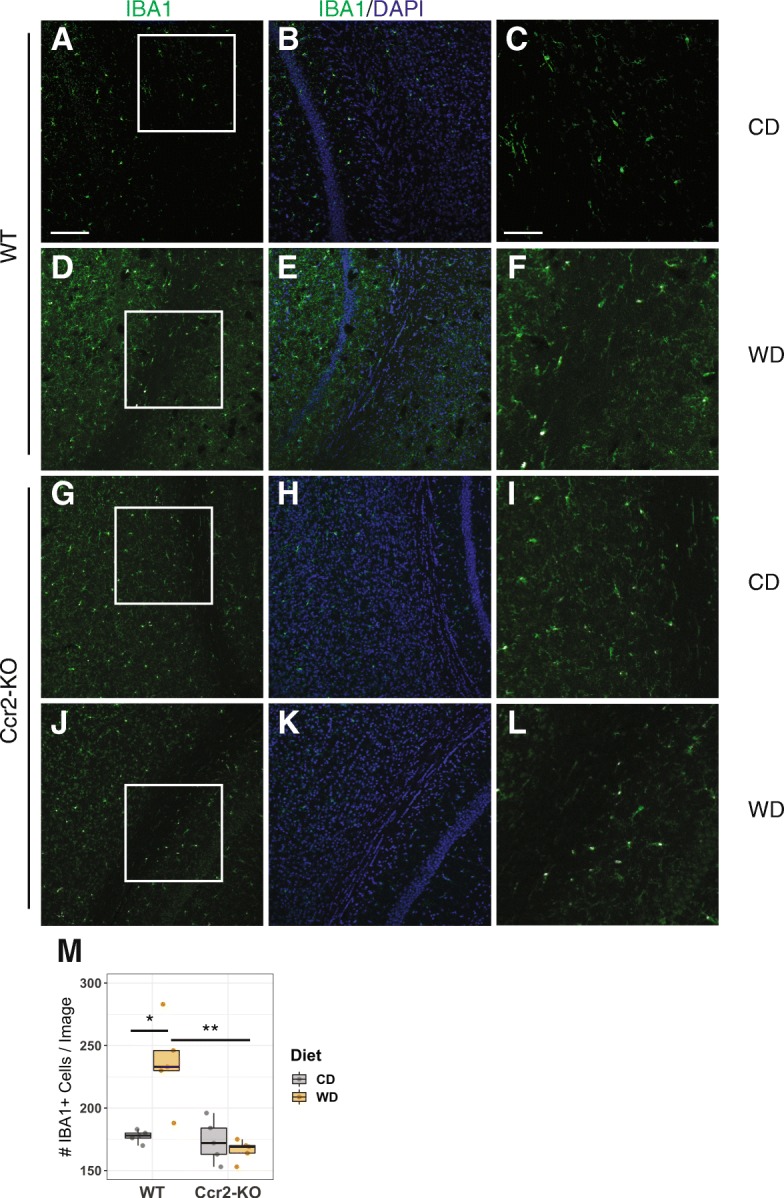
CCR2 deficiency inhibited the WD-induced increase in IBA1+ cells. **a**–**l** Representative cortical sections of IBA1 staining from 12-month-old WT (**a**–**f**) and *Ccr2*-KO mice (**g**–**l**) under CD or WD. **c**, **f**, **i,** and **l** are magnified images from boxed region shown on **a**, **d**, **g**, and **j**, respectively. **m** The average number of IBA1+ cells per image (averaged across at least three images) increased in WT mice but not in *Ccr2*-KO mice (two-way ANOVA followed by Tukey HSD post hoc test, *p < 0.05, **p < 0.01). Scale bars: **a**, **b**, **d**, **e**, **g**, **h**, **j**, and **k**, 100 μm; **c**, **f**, **i**, and **l**, 40 μm

To further assess the contribution of CCR2 to myeloid cell numbers in WD-fed mice, *Ccr2*-KO and WT controls were fed either a WD or CD from 2 to 12 months. Brains were harvested, fixed, sectioned, and labeled with IBA1 (a commonly used marker of microglia, monocytes, and macrophages) and DAPI (a nuclear marker). IBA1+DAPI+ cell number was determined in WD- and CD-fed mice of both genotypes (Fig. 2). As expected, the number of IBA1+DAPI+ cells was significantly greater in WD-fed WT mice compared to CD-fed WT mice. However, CCR2 deficiency blunted the effect of a WD-induced increase in IBA1+DAPI+ cells. This further supports the hypothesis that an increase in the total number of brain myeloid cells correlates to an increased number of infiltrating peripheral myeloid cells in response to a WD.

### The percent of CD11b+CD45^hi^ myeloid cells increased in the brains of WD-fed mice in a CCR2-dependent manner

**Fig. 3:**
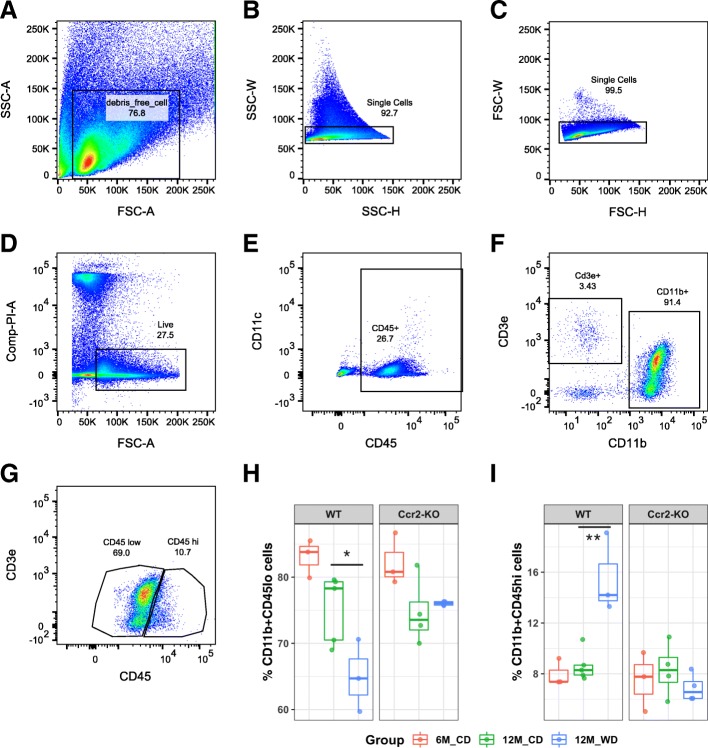
CCR2 deficiency inhibited the WD-induced increase in CD11b+CD45^hi^ cells. **a**–**g** Gating strategy to isolate CD11b+CD45^lo^ and CD11b+CD45^hi^ cells by FACS. Forward scatter (FSC) and side scatter (SSC) were used to remove the cell debris and select single cells (**a**–**c**). All live cells, the propidium iodide (PI) negative population, were then selected for immune cell marker profiling (**d**). CD45+ cells were selected (**e**) and were subdivided into CD11b–CDe3+ cells and CD11b+CD3e− cells (**f**). CD11b+CD3e− cells were further subdivided into CD11b+CD45^lo^ and CD11b+CD45^hi^ cells (**g**). Representative gating images were from a 12-month B6 mouse fed a CD. **h** The percent of CD11b+CD45^lo^ (**g**) from total CD11b+CD45+ cells (**f**). **i** The percent of CD11b+CD45^hi^ cells (**g**) from total CD11b+CD45+ cells (**f**). Brain samples were from WT or *Ccr2*-KO mice of 6-month fed a CD, or 12-month fed a CD or WD (two-way ANOVA followed by Tukey HSD post hoc test, *p < 0.05, **p < 0.01)

Myeloid cells in the brain have been characterized previously by the presence of CD11b and differential levels of CD45. CD11b+CD45^hi^ cells are considered to be enriched for infiltrating cells, while CD11b+CD45^lo^ cells represent resident microglia [18]. Therefore, to characterize brain myeloid cells after WD consumption, these two subpopulations of myeloid cells were assessed by flow cytometry, FACS, and RNA-sequencing. Brains from WT or *Ccr2*-KO mice fed either a WD or CD from 2 to 12 months, or CD from 2 to 6 months, were collected and dissociated into a single-cell suspension. Cells were stained with various immune cell markers including CD45, CD11b, CD11c, CD3e, Ly6c, and Ly6g for flow cytometry and FACS. After selecting live single cells **(**Fig. 3a–d**)**, both CD11b-positive and CD45-positive cells **(**Fig. 3e, f**)** were subdivided into CD11b+CD45^lo^ and CD11b+CD45^hi^ cells **(**Fig. 3g**)**. Importantly, the percent of CD11b+CD45^hi^ cells was significantly increased (*p* = 0.0013) with a corresponding percent decrease of CD11b+CD45^lo^ cells (*p* = 0.041) in 12-month WD-fed mice compared to 12-month CD-fed WT mice (Fig. 3h, i). No significant increase was observed in *Ccr2*-KO mice fed a WD **(**Fig. 3i**)**.

As CD11c (encoded by *Itgax*) can be a marker for dendritic cells (reviewed in [30]) and/or potential disease-associated microglia (DAM) [31], we also characterized CD45+CD11c+ cells (Additional file 1: Figure S1A). The percent of CD45+CD11c+ cells was also increased in 12-month mice fed a WD compared to its CD-fed counterparts, and this effect is blunted by CCR2 deficiency (Additional file 1: Figure S1D). CD11c was also predominately expressed in a subset of CD11b+CD45^hi^ cells **(**Additional file 1: Figure S1B-C**)**; the percent of CD45^hi^CD11c+ cells was significantly higher than that of CD45^lo^CD11c+ cells **(**Additional file 1: Figure S1E**)**. Furthermore, we used a combination of Ly6c and Ly6g markers to mark monocyte-like cells (CD11b+CD45+Ly6c+Ly6g–) and granulocyte-like cells (CD11b+CD45+Ly6c+Ly6g+) (Additional file 1: Figure S2A). The percent of total Ly6c+Ly6g− cells was significantly lower in *Ccr2*-KO mice than in WT mice (Additional file 1: Figure S2D). However, the percent of total Ly6c+Ly6g+ cells was not significantly different between WT and *Ccr2*-KO mice (Additional file 1: Figure S2F). Moreover, both Ly6c+Ly6g− cells (*p* = 1.99e−9) and Ly6c+Ly6g+ cells (*p* = 2.58e−6) are significantly enriched in CD11b+CD45^hi^ cells compared to CD11b+CD45^lo^ cells (Additional file 1: Figure S2B, C, E, G). Interestingly, the percent of T cells, the CD45+CD11b-CD3e+ population (Fig. 3f), was also significantly increased in 12-month CD-fed WT mice compared to 6-month counterparts (*p* = 0.00088), but not in *Ccr2*-KO mice (Additional file 1: Figure S3).

Together, our data show that CD11b+CD45^hi^ myeloid cells were increased in the brains of WD-fed mice in a CCR2-dependent manner. CD11b+CD45^hi^ cells are composed of CD11c+, Ly6c+Ly6g−, and Ly6c+Ly6g+ cells that may represent a variety of myeloid cell subtypes including activated microglia and infiltrating inflammatory monocyte- or granulocyte-like cells.

### Characterizing the transcriptomics of CD11b+CD45^lo^ and CD11b+CD45^hi^ myeloid cells in the brains of WD-fed mice

Given their similarities, it is currently challenging to isolate, characterize, and functionally test subpopulations of myeloid cells in obese brains. Therefore, to identify genes and pathways relevant to myeloid cell responses, CD11b+CD45^lo^ and CD11b+CD45^hi^ cells from 12-month B6 mice fed either a WD or CD were collected by FACS and profiled by RNA-seq (Fig. 3g). The purity of myeloid cell populations was validated by previously described cell type-specific markers in the brain (Additional file 1: Figure S4). Both *Itgam* (that encodes CD11b) and *Ptprc* (that encodes CD45) are highly enriched in CD11b+CD45^lo^ and CD11b+CD45^hi^ cells. As expected, the level of *Ptprc* in CD11b+CD45^hi^ cells was 2.23 (FDR = 1.80e−3) and 2.93 (FDR = 1.76e−5) fold higher than CD11b+CD45^lo^ cells in CD or WD-fed mice, respectively (Additional file 1: Figure S4). There was no detectable level of *Rbfox3* (NeuN, neuronal marker) and negligible levels (average cpm < 10) of *Aldh1l1* (astrocyte marker), *Pdgfrb* (pericyte marker), Mcam (endothelial cell marker), and *Mog* (oligodendrocyte marker) in both CD11b+CD45^lo^ and CD11b+CD45^hi^ cell populations from either CD- or WD-fed mice **(**Additional file 1: Figure S4). Together, these data validated the high purity of FACS-enriched myeloid cell populations.

**Fig. 4:**
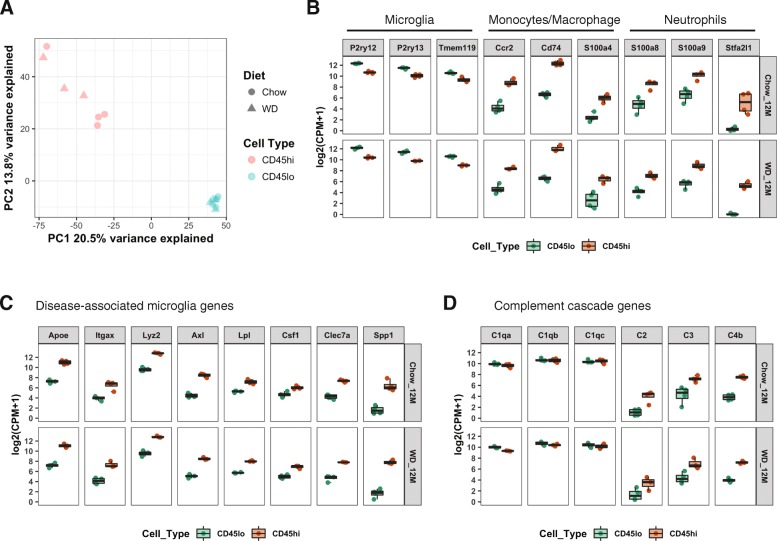
Characterization of DE genes in CD11b+CD45^lo^ and CD11b+CD45^hi^ cells of CD and WD-fed mice. **a** Principal component analysis (PCA) plot showing the first and second component of transcriptional expression profiles between CD11b+CD45^lo^ and CD11b+CD45^hi^ cells. **b**–**d** Box plots showing the gene expression in log_2_-transformed cpm in CD11b+CD45^lo^ and CD11b+CD45^hi^ cells from both CD and WD-fed mice. **b** Expression of marker genes of microglia, monocytes/macrophage, and granulocytes. **c** Expression of disease-associated microglia (DAM) genes. **d** Expression of complement cascade genes

Principal component analysis (PCA) showed striking differences in the transcriptomes between CD11b+CD45^lo^ and CD11b+CD45^hi^ cells isolated from mice fed either a WD or CD. These cell populations were separated by the first and second principal components (PC1 and PC2) which explained 20.5% and 13.8% variance in their transcriptome profiles, respectively (Fig. 4a). Consistent with the PCA, 740 DE genes (FDR < 0.05 and absolute FC > 1.5) resulted from pairwise comparison of CD11b+CD45^lo^ and CD11b+CD45^hi^ cells from WD-fed mice (Additional file 2) and 554 DE genes (FDR < 0.05 and absolute FC > 1.5) from pairwise comparison of CD11b+CD45^lo^ and CD11b+CD45^hi^ cells from CD-fed mice (Additional file 3). However, there were no DE genes comparing transcriptomes of the same cell type (CD11b+CD45^lo^ or CD11b+CD45^hi^ cells) between WD and CD (FDR < 0.05 and absolute FC > 1.5). This agreed with the PCA which showed no clear separation between samples from mice fed different diets. This is likely because, despite significant differences in the percent of either CD11b+CD45^lo^ or CD11b+CD45^hi^ cells between WD- and CD-fed mice as measured by flow cytometry (Fig. 3h, i), the numbers of cells collected for transcriptional profiling were normalized across samples. PC2 did suggest subtle variation in CD11b+CD45^hi^ cells between WD and CD samples (Fig. 4a). To determine the genes driving this potential subtle difference, a reduced stringency (absolute FC > 1.0) was employed and 87 DE genes were identified comparing CD11b+CD45^hi^ samples between mice fed a CD and WD (Additional file 4). No DE genes were detected using this lower stringency for CD11b+CD45^lo^ cells. The 87 DE genes were subjected to the canonical pathway function in Ingenuity Pathway Analysis (IPA) software to identify pathways enriched in these genes. The top canonical pathway identified is protein ubiquitination pathway (Additional file 1: Figure S5A) that includes DE genes *Psmb9*, *Usp10*, *Dnajc1*, *Sugt1*, and *Ube2w* (Additional file 1: Figure S5B, C). Interestingly, *Ctsd*, the gene encoding cathepsin D which is a critical lysosomal enzyme associated with lipid metabolism and obesity [32, 33], was significantly increased in CD11b+CD45^hi^ cells of WD-fed mice comparing that of CD-fed mice (FC = 1.38) (Additional file 1: Figure S5B, C). This suggests that compared to CD, WD may have a subtle effect on the metabolic function of CD11b+CD45^hi^ cells.

To further characterize genes and pathways that define the differences between CD11b+CD45^lo^ and CD11b+CD45^hi^ cells, the expression levels of several classical myeloid cell markers were assessed. CD11b+CD45^lo^ cells expressed significantly higher levels of microglia signature genes including *P2ry12*, *P2ry13*, and *Tmem119* compared to CD11b+CD45^hi^ cells **(**Fig. 4b**)**. However, CD11b+CD45^hi^ cells also expressed these signature genes (albeit at lower levels to CD11b+CD45^lo^ cells) suggesting that the CD11b+CD45^hi^ population also includes microglia. Other studies have suggested that using differential levels of CD45 is not a definitive method to define and then isolate resident microglia and peripheral myeloid cells [18], and our data support this. Interestingly, DAM genes (including *Itgax*) that were defined in a recent single-cell sequencing study [31] were more highly expressed in CD11b+CD45^hi^ cells compared to CD11b+CD45^lo^ cells suggesting CD11b+CD45^hi^ cells may be enriched for activated or “responding” microglia while CD11b+CD45^lo^ cells are primarily resting or “sensing” microglia **(**Fig. 4c**)**. This result is also supported by our flow cytometry data that showed the majority of CD11c+ cells were present in CD11b+CD45^hi^ cells (Additional file 1: Figure S1E).

To determine whether CD11b+CD45^lo^ or CD11b+CD45^hi^ cells are enriched for peripheral myeloid cells, classical markers of monocytes/macrophages and granulocytes were assessed. The monocyte/macrophage genes *Ccr2*, *Cd74*, and *S100a4* were more highly expressed in CD11b+CD45^hi^ compared with CD11b+CD45^lo^ cells (Fig. 4b). Similarly, the granulocyte genes *S100a8*, *S100a9*, and *Stfa2l1* were highly expressed in CD11b+CD45^hi^ cells, with *Stfa2l1* as the newly identified neutrophil marker [34]. This result was also supported by our flow cytometry data that showed more Ly6c+Ly6g− and Ly6c+Ly6g+ cells were present in CD11b+CD45^hi^ cells (Additional file 1: Figure S2E, G). Together, our data support our model that CD11b+CD45^hi^ cells are enriched for infiltrating peripheral myeloid cells. Interestingly, both CD11b+CD45^lo^ and CD11b+CD45^hi^ cells expressed high levels of C1q complex-coding genes (*C1qa*, *C1qb*, and *C1qc*) (Fig. 4d), while CD11b+CD45^hi^ cells expressed significantly higher levels of downstream complement components (*C2*, *C3*, and *C4b*) than CD11b+CD45^lo^ cells (Fig. 4d), suggesting the complement cascade, a component of the innate immune response, is more active in CD11b+CD45^hi^ cells compared to CD11b+CD45^lo^ cells.

### Pathway analyses identified enrichment of genes involved in diapedesis in CD11b+CD45^hi^ cells in obesity, aging, and AD

**Fig. 5:**
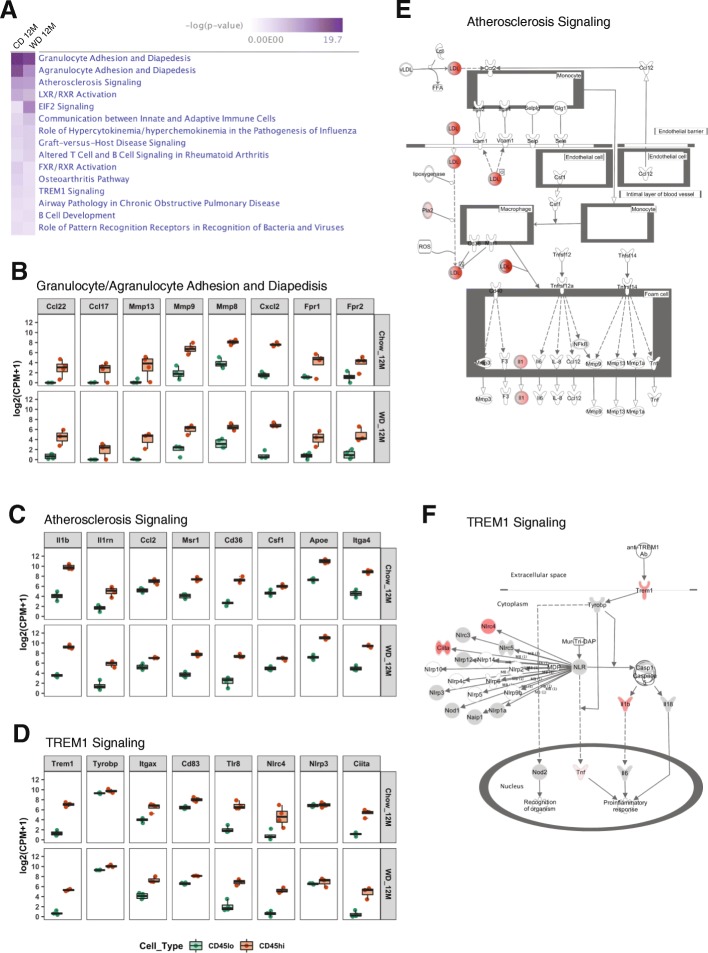
Ingenuity Pathway Analysis (IPA) of DE genes between CD11b+CD45^lo^ and CD11b+CD45^hi^ cells. **a** IPA reveals the top 15 canonical pathways based on the DE genes comparing CD11b+CD45^lo^ and CD11b+CD45^hi^ cells in CD or WD diet-fed mice [−log_10_(*p* value) > 1.3]. **b**–**d** Box plots showing the expression levels of the representative genes in CD11b+CD45^lo^ and CD11b+CD45^hi^ cells in granulocyte or agranulocyte adhesion and diapedesis (**b**), atherosclerosis signaling (**c**), and TREM1 signaling pathways (**d**). **e**–**f** Pathway network illustration of atherosclerosis signaling (**e**) and TREM1 signaling (**f**) pathways in WD-fed mice. The molecules in red indicate their significantly increased expression in CD11b+CD45^hi^ cells compared to CD11b+CD45^lo^ cells

A major goal of this study was to determine genes/pathways to help define the function of cells in the CD11b+CD45^hi^ population. Therefore, we employed the canonical pathway function in IPA software to identify pathways enriched in the 740 and 554 DE genes from WD and CD, respectively, comparing the CD11b+CD45^hi^ samples to the CD11b+CD45^lo^ samples **(**Additional files 2 and 3). As expected, most of the top 15 canonical pathways were involved in immune-related functions (Fig. 5a). Importantly, the top two pathways are granulocyte or agranulocyte adhesion and diapedesis (Fig. 5a, b), suggesting CD11b+CD45^hi^ cells include peripheral myeloid cells as a result of their extravasation into the brain. Atherosclerosis signaling was the third significant pathway (Fig. 5a, c, and e) suggesting CD11b+CD45^hi^ cells contribute to chronic cerebrovascular inflammation that may result from the build-up of fatty material under chronic WD consumption. Another significant pathway was TREM1 signaling (Fig. 5a, d, and f) suggesting that CD11b+CD45^hi^ cells mediate proinflammatory responses in the brain. Together, our pathway analyses further support our model that CD11b+CD45^hi^ cells are composed of peripheral monocytes and granulocytes that infiltrate into the brain and contribute to WD-related neuroinflammation.

While aging is the greatest risk factor for AD, obesity also significantly increases risk. Further, multiple lines of evidence show that myeloid cells may be central to brain changes observed in obesity, aging, and AD [9–13]. Therefore, to determine the similarities and differences between myeloid cell populations in WD-fed mice, AD mice, and aging mice, transcriptional profiles were generated from CD11b+CD45^lo^ and CD11b+CD45^hi^ cells isolated from B6 mice fed the CD from 2 to 20 months, and B6.*APP/PS1* mice fed the CD from 2 to 6 months (early plaque deposition in this strain). Interestingly, the DE genes comparing transcriptional profiles between CD11b+CD45^lo^ and CD11b+CD45^hi^ cells in aging and AD showed similar enriched pathways to those identified in WD-fed mice (Additional file 1: Figure S6).

### Top genes enriched in CD11b+CD45^hi^ cells reflected peripheral myeloid cell profiles

**Fig. 6:**
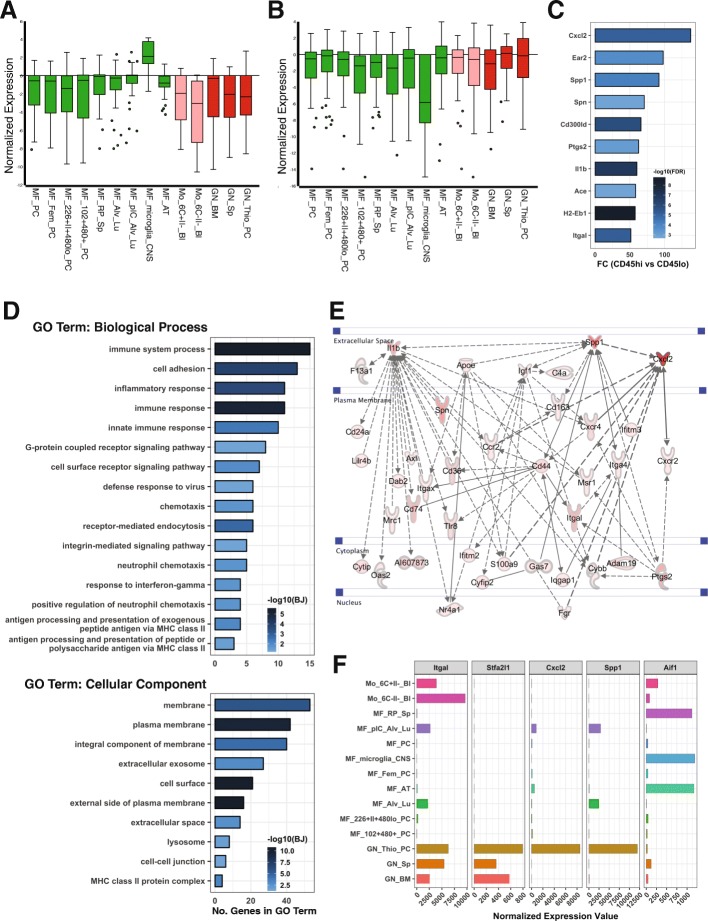
Top genes enriched in CD11b+CD45^hi^ cells reflected peripheral myeloid cell profiles in WD-fed mice. **a**, **b** Normalized gene expression plot (reproduced from ImmGen datasets, see the “Methods” section) showing relative gene expression values for 34 CD11b+CD45^lo^ cell-enriched DE genes (**a**) or 73 CD11b+CD45^hi^ cell-enriched DE genes (**b**) within macrophage (green), monocytes (pink), and neutrophils (red). Normalized expression means log_2_(gene expression value/average expression value of all genes). ImmGen immune cell type abbreviations: MF = macrophage, Mo = monocytes, GN = neutrophils, PC = peritoneal cavity, Bl = blood, Sp = spleen, BM = bone marrow, Alu_Lu = alveolar cell in the lung, Fem = female, CNS = central nervous system. **(c)** Fold change (FC) of top 10 CD11b+CD45^hi^ cell-enriched genes (FC compared to CD11b+CD45^lo^ cells) in WD-fed mice, colored by the significance level using -log_10_(FDR). **d** Numbers of genes involved in GO term of Biological Process and cellular component analysis based on 73 CD11b+CD45^hi^ cell-related genes in WD-fed mice, colored by significance level using -log_10_(BJ). **e** Gene interaction network generated in IPA containing 37 of the 73 CD11b+CD45^hi^ cell-enriched genes in WD-fed mice. **f** Normalized expression levels of *Itgal* (blood-derived immune cell marker), *Cxcl2*, *Spp1*, *Stfa2l1* (specific neutrophil marker), and *Aif1* (brain myeloid cell marker) across macrophage, monocytes, and granulocytes reproduced from ImmGen datasets (same datasets as **a** and **b**)

Data presented here and previous data suggest that CD11b+CD45^hi^ cells are a mixed population that includes potentially responding (activated) brain-resident microglia and peripheral myeloid cells that infiltrated into the brain after WD consumption. To determine their respective contributions to brain health, it is essential to determine genes that define these different myeloid subpopulations to enable functional testing. To identify gene sets that better define these subpopulations, we set stringent criteria to determine the top DE genes enriched in CD11b+CD45^hi^ and CD11b+CD45^lo^ cells (detailed in the “Methods” section). This strategy allowed us to identify genes that showed high levels of expression in one cell population and negligible expression in the other cell population. Using this approach, we identified a set of 34 genes that are enriched in CD11b+CD45^lo^ cells (Additional file 5) and a set of 73 genes highly enriched in CD11b+CD45^hi^ cells in the brains of WD-fed mice (Additional file 6). To classify the myeloid cell subtypes present in the CD11b+CD45^lo^ and CD11b+CD45^hi^ populations, both gene sets were compared against the RNA-sequencing database of all immune cell types deposited at ImmGen [25]. The 34 CD11b+CD45^lo^ cell-related genes specifically matched the microglia profile, but not any other tissue macrophages, monocytes, or neutrophils (Fig. 6a). In contrast, the 73 CD11b+CD45^hi^ cell-related genes more accurately matched to monocytes and neutrophils with the least alignment against the microglia profile (Fig. 6b). The five most highly enriched genes based on fold change (CD11b+CD45^hi^ compared to CD11b+CD45^lo^) were *Cxcl2* (137.63-fold), *Ear2* (97.96-fold), *Spp1* (91.95-fold), *Spn* (70.74-fold), and *Cd300ld* (65.97-fold) (Fig. 6c). The seventh most enriched gene was *Il1b* (60.14-fold), a proinflammatory marker. The tenth most enriched gene was *Itgal* that codes for CD11a, a protein widely used as a marker of peripheral immune cells [35] (Fig. 6f). As expected given the sorting strategy (Fig. 3), neither gene set matched well with other immune cell types including B cells, T cells, dendritic cells, NK cells, mast cells, basophils, or stromal cells (Additional file 1: Figure S7).

To predict the functions of the CD11b+CD45^hi^ cell-related genes, Gene Ontology (GO) enrichment analysis was performed on the 73 genes. Enriched biological process (BP) GO terms related to immune-related processes (Fig. 6d). The second enriched BP GO term was cell adhesion, further supporting that CD11b+CD45^hi^ cell is involved in diapedesis, as cell adhesion occurs before diapedesis (Fig. 6d). Interestingly, enriched BP GO terms also included neutrophil chemotaxis and positive regulation of neutrophil chemotaxis, suggesting that the CD11b+CD45^hi^ cells could include neutrophils, the major cell subtype of granulocytes. GO term analysis was also consistent with pathway analysis in IPA that revealed granulocyte adhesion and diapedesis as the top canonical pathway (Fig. 5a). Furthermore, the cellular component (CC) GO term enrichment analysis suggested CD11b+CD45^hi^ cell-related genes include genes encoding membrane-bound or extracellular proteins (Fig. 6d). Using the gene interaction network function of IPA, 37 of the 73 genes appeared in a single gene network (Fig. 6e), including *Cxcl2*, *Spp1*, *Spn*, *Ptgs2*, *Il1b*, and *Itgal*, that were among the top 10 CD11b+CD45^hi^ cell-related genes (Fig. 6c). Consistent with CC GO term enrichment, proteins encoded by the CD11b+CD45^hi^ cell-related genes in this network are highly enriched in plasma membrane component, serving as immune cell surface receptors.

To further refine which cell type the GO terms and gene network may be functioning in, key genes in the network were compared to the ImmGen datasets [26]. Two of the most highly enriched genes in this network, *Cxcl2* and *Spp1*, were predominantly expressed in thioglycolate broth (TG) stimulated neutrophils, showing a strikingly similar expression pattern to the recently identified neutrophil marker, *Stfa2l1* [34] (Fig. 6f). Together, these data suggest that the CD11b+CD45^hi^ cell population included a significant number of neutrophils.

### Chronic western diet consumption induced OPN-expressing cells in the brain

GO term and gene network analyses predicted that the gene network containing *Cxcl2* and *Spp1* is functioning in activated neutrophils. *Spp1* encodes for osteopontin (OPN), a proinflammatory cytokine that has been shown to be secreted from numerous cells including activated leukocytes [36]. However, ImmGen data suggests that stimulated neutrophils are the greatest producer of OPN (Fig. 6f). Interestingly, in the ImmGen datasets, *Spp1* was not expressed in microglia that did express high levels of *Aif1* (that encodes IBA1, a commonly used marker for microglia and monocytes/macrophages) (Fig. 6f). *Aif1* was not expressed by stimulated neutrophils that highly express *Spp1* (Fig. 6f).

**Fig. 7:**
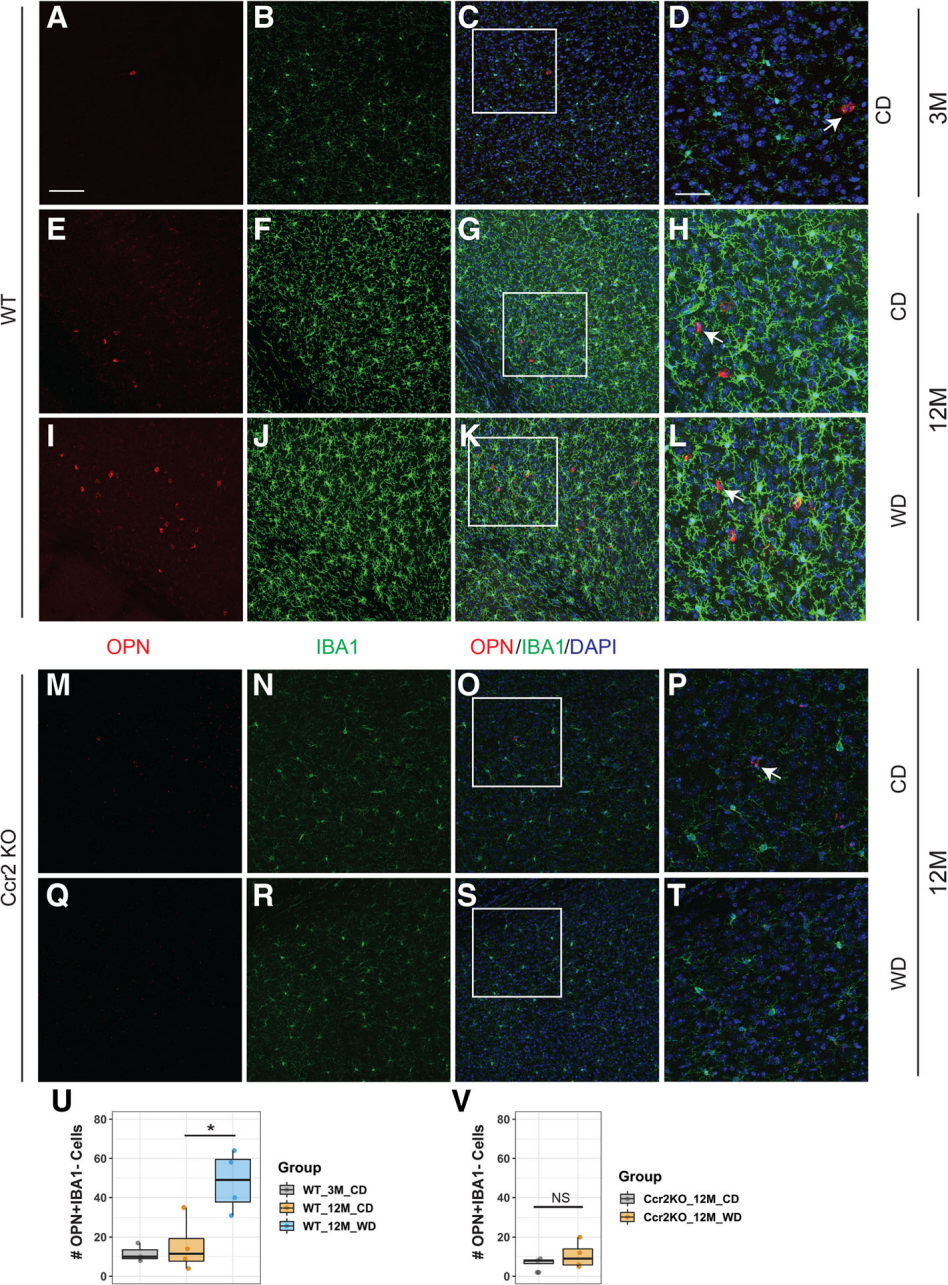
WD increased OPN+IBA1− cells in the brain. **a**–**l** Representative cortical sections on OPN (red) and IBA1 (green) staining from 3-month WT mice fed a CD (**a**–**d**), 12-month-old WT mice fed a CD (**e**–**h**) or WD (**i**–**l**). **m**–**t** Representative cortical sections on OPN (red) and IBA1 (green) staining from 12-month *Ccr2*-KO mice fed a CD (**m**–**p**), or WD (**q**–**t**). **d**, **h**, **l**, **p**, and **t** are magnified images cropped from **c**, **g**, **k**, **o**, and **s**, respectively. Arrows indicate examples of OPN+IBA1− cell in each group. **u** Box plots showing the number of OPN+IBA1− cells (sum of cell numbers from seven images per animal) in 3-month WT CD-fed mice, and 12-month CD or WD-fed WT (one-way ANOVA followed by Tukey HSD post hoc test, *p = 0.016). **v** Box plots showing the number of OPN+IBA1− cells (sum of cell numbers from seven images per animal) in 3-month *Ccr2*-KO and 12-month CD or WD-fed WT (*T* test; not significant, NS). Scale bars: **a**–**c**, **e**–**g**, **i**–**k**, **m**–**o**, and **q**–**s**, 100 μm

To further characterize *Spp1*, OPN and IBA1 immunoreactivity was examined in young (3 months) and aged (12 months) WT mice fed a CD or WD and 12-month *Ccr2*-KO mice fed a CD or WD. There was a significant increase in OPN+IBA1− cells in aged WT WD-fed mice compared to their aged CD-fed counterparts (Fig. 7e–l, u). However, this effect was not observed in *Ccr2*-deficient mice (Fig. 7m–t, v). Additionally, there was no significant difference in OPN+IBA1− cell number comparing young and aged mice under CD (Fig. 7a–h, u). There was a significant decrease in OPN+IBA1+ cells in 12-month WT WD-fed mice compared to WT CD-fed mice (Additional file 1: Figure S8A). However, there was no significant difference between the number of OPN+IBA1+ cells in aged *Ccr2*-KO mice under CD vs. WD (Additional file 1: Figure S8B). Together, our data suggest that a population of OPN+IBA1− cells enter the brain in response to a WD via a CCR2-dependent mechanism. We speculate this OPN+IBA1− population likely represents activated neutrophils.

## Discussion

Brain myeloid cells are well recognized in WD- and obesity-induced neuroinflammation. However, to our knowledge, this study is the first to perform transcriptomic characterization of brain myeloid cell subpopulations in obese mice. We characterized two distinct subsets of brain myeloid cells, CD11b+CD45^lo^ and CD11b+CD45^hi^ cells in mice fed a WD from 2 to 12 months. Our flow cytometry, FACS, and transcriptomic analyses showed that CD11b+CD45^lo^ cells are most likely the steady-state brain-resident microglia while CD11b+CD45^hi^ cells are likely composed of responding (or activated) microglia, dendritic cells and peripherally derived cells (monocytes/macrophages and neutrophils). The heterogeneity of CD11b+CD45^hi^ cells in our study is likely due to the fact that CD45 is not categorical to separate different subpopulations of myeloid cells—particularly brain-resident vs. peripherally derived—at least in the context of neuroinflammation induced by chronic conditions like obesity, as our flow cytometry data showed continuous levels of CD45 expression across CD11b+CD45+ populations. In support of this, we detected common microglia markers such as *Tmem119*, *P2ry12*, and *P2ry13* in transcriptomes from CD11b+CD45^hi^ cells. Therefore, improved markers for distinguishing peripheral myeloid cell subpopulations from resident microglia are needed to determine the function of these different cell populations in health and disease.

Our flow cytometry and FACS data showed that chronic WD consumption increased the percent of CD11b+CD45^hi^ cells, likely through a CCR2-dependent mechanism. This suggests that certain subpopulations of CD11b+CD45^hi^ cells such as monocytes and neutrophils may be increased in the brain during WD. This is also supported by our observation of the increase in IBA1+ cells and OPN+ cells in WD-fed mice compared to CD-fed mice. However, no significant differences in Ly6c+Ly6g+ or Ly6c+Ly6g– cells were detected, and this may be due to the reported down-regulation of these markers upon entry into tissue (reviewed in [30]). Our data showed that some CD11b+CD45^hi^ cells are present in young and aging brains. This suggests immune surveillance, whereby peripheral cells enter the brain, is occurring at low levels in young and healthy aging brains [37, 38], and this process increases in response to a WD. Interestingly, we found that the percent of T cells increased in an age- and CCR2-dependent manner. It is possible that peripheral T cells infiltrate into the brain during aging [39] and potentiate or contribute to neuroinflammation through interaction with myeloid cells during neuropathological conditions [40]. The increased WD-associated neuroinflammation may mostly be explained by the increased number of peripheral myeloid cells and activated microglia presented in CD11b+CD45^hi^ cells, rather than altered gene expression in either CD11b+CD45^lo^ cells or CD11b+CD45^hi^ cells by WD. We detected no effect of WD on transcriptomes of CD11b+CD45^lo^ cells and possible subtle effects on transcriptomes of CD11b+CD45^hi^ cells. However, this observation could also be limited by our current technology. The gene expression of microglia or infiltrating myeloid cells during aging and/or WD consumption may show some brain region-specific responses [8, 39, 41]. Therefore, such potential region-specific gene expression pattern of CD11b+CD45^lo^ or CD11b+CD45^hi^ cells may be undetectable when myeloid cells from whole cerebral tissue were sequenced. Further studies incorporating spatial single-cell transcriptomic technologies are necessary [42–44].

By comparing transcriptional profiles from CD11b+CD45^hi^ cells compared to CD11b+CD45^lo^ cells in WD-fed mice, we identified 740 DE genes, 73 of which were almost exclusively expressed in CD11b+CD45^hi^ cells. The 73 genes included common markers of peripheral myeloid cells including *Itgal* (that encodes CD11a). Pathway analyses of the DE genes comparing CD11b+CD45^hi^ cells with CD11b+CD45^lo^ cells revealed enrichment of genes involved in granulocyte and agranulocyte adhesion and diapedesis, and atherosclerosis signaling (Fig. 5a). These closely related pathways imply that in WD-fed mice, peripheral monocytes, and neutrophils may interact with the cerebrovascular system leading to vascular dysfunction [45, 46]. Similar to an atherogenic condition, WD consumption can also cause dysregulation of lipid and cholesterol metabolism and mediate proinflammatory responses. Together, these lead to disruption of blood-brain barrier (BBB) homeostasis, causing increased BBB permeability via activation of endothelial cells and pericytes, key components and regulators of BBB [47]. Upon activation of WD-induced immune stimuli, endothelial cells can produce proinflammatory cytokines to attract peripheral myeloid cells including monocytes and neutrophils to the “injury” site [48–51]. Pericytes can also be activated to release proinflammatory cytokines or reactive oxygen species that further exacerbate BBB disruption by destabilizing tight junction proteins [52]. Together, these events can facilitate the transmigration of peripheral myeloid cells into the brain parenchyma. These peripheral myeloid cells can then amplify local inflammatory responses by producing proinflammatory cytokines such as IL-1, IL-6, and MMPs, which in turn cause additional cerebrovascular damage. For example, MMP9 has been shown to degrade basement membrane allowing monocytes and neutrophils to enter perivascular space causing BBB decomposition [53–55]. In our study, *Mmp9* is highly enriched in CD11b+CD45^hi^ compared to CD11b+CD45^lo^ cells (Fig. 5b). BBB integrity is crucial for maintaining normal brain functions; breakdown allows entry of neurotoxic blood-derived cells, metabolites, and pathogens that initiate neurodegenerative processes [47]. Together, the pathway analyses support a model whereby WD-induced obesity leads to BBB leakage through the interaction of peripheral myeloid cells and vascular cells such as endothelial cells and pericytes.

Gene expression analysis showed CD11b+CD45^hi^ cells expressed high levels of *Trem1* (FC = 60.10 compared to CD11b+CD45^lo^ cells, Fig. 5d) suggesting they exhibit robust TREM1 signaling. TREM1 is selectively expressed on neutrophils and monocytes [56]. As a potent amplifier of acute and chronic inflammation, TREM1 has been linked with obesity, atherosclerosis, and AD. TREM1 expression was significantly increased in monocytes and neutrophils from blood, adipose, and liver biopsies in obese individuals [57]. In a second study, the expression of TREM1 was also significantly increased along with the proinflammatory M1 markers in liver biopsies of obese patients [58]. Genetic and pharmacological inhibition of TREM1 ameliorated atherosclerosis in mice [59]. Recent studies have also implicated TREM1 in AD. Protein quantitative trait analysis of human monocytes revealed that the rs6910730G variant in the *TREM1* locus was associated with a decreased *TREM1*/*TREM2* ratio and increased pathological features of AD and aging-related cognitive decline [60]. Another study suggested TREM1 facilitated microglial Aβ phagocytosis while the rs6910730G variant impaired this function and exacerbated AD pathogenesis [61]. These studies suggest TREM1 may play a beneficial role in Aβ clearance. However, TREM1 is highly expressed in neutrophils [56]. A recent study demonstrated that neutrophils transmigrated through the BBB into the AD mouse brain, worsening the AD phenotype [62]. In contrast, neutrophil depletion improved memory and reduced AD-relevant pathology [62]. TREM1 has also been implicated in vascular dysfunction [57–59], so the role of TREM1 in brain health is likely complex. Much attention is currently being given to *TREM2* in AD and other neurodegenerative diseases [63, 64]. However, given the complexity of neuroinflammatory responses in aging brains, other members of the TREM family (particularly TREM1) require more consideration.

Our stringent analysis of DE genes identified *Spp1* (that encodes OPN) as predominantly enriched in CD11b+CD45^hi^ cells and OPN+IBA1− cells were increased in WD-fed compared to CD-fed mice. As a proinflammatory cytokine, OPN is well recognized for controlling immune cell functions such as recruitment of monocytes/macrophage and facilitating cytokine secretion in leukocytes [65]. Studies suggest that OPN is critically involved in inflammation in adipose tissue during diet-induced obesity. In high-fat diet-fed mice, OPN was increased dramatically in macrophages that were recruited to adipose tissue [66], promoting extracellular matrix remodeling as well as proinflammatory responses [67]. OPN deficiency blocked macrophage infiltration into adipose tissue, disrupted extracellular matrix remodeling and reduced inflammation [66–68]. However, the role of OPN in the brain during diet-induced obesity is less known. We showed that OPN+IBA1− cells but not OPN+IBA1+ cells increased in the brain of WD-fed mice in a CCR2-dependent manner. This is consistent with the observation that GFP+RFP+ cells (RFP expression driven by the *Ccr2* promoter) increase in response to the WD (Fig. 1i). However, the exact relationship between cells expressing *Cx3cr1*::GFP, *Ccr2*::RFP, OPN, and IBA1 remains to be elucidated. Our result suggests that the increase in OPN-expressing cells is likely as a result of infiltration of peripheral myeloid cells, particularly neutrophils. In support of this, *Spp1* is primarily expressed in activated neutrophils based on the ImmGen transcriptional profiling data (Fig. 6f). *Spp1* was expressed at a minimal level in unstimulated bone marrow and spleen neutrophils suggesting *Spp1* is not expressed in steady-state neutrophils but may be dramatically upregulated upon stimulation, such as WD-induced inflammation. Although CCR2 is largely considered a marker for peripheral monocytes, CCR2 is also expressed in other immune cell types including neutrophils, albeit at a lower level [69]. CCR2 deficiency not only blocked monocyte infiltration into the brain, but also inhibited infiltration of neutrophils [69–71], as CCR2 deficiency sequesters multiple subsets of leukocytes including neutrophils in the bone marrow [69]. Collectively, these studies support our model that the OPN levels in brains of WD-fed mice are mediated by CCR2-dependent infiltration of neutrophils. However, the specific function of OPN is not well understood. In one study, OPN colocalized with CD68-positive myeloid cells in vessels with an impaired BBB in stroke-prone spontaneously hypertensive rats, indicating OPN-expressing microglia or macrophages may be involved in regulating BBB homeostasis [72]. Other studies suggested OPN may be protective to BBB after subarachnoid hemorrhage [73, 74]. Whether OPN is beneficial or detrimental to BBB and overall brain health during WD-induced obesity and other brain disorders remains to be determined.

## Conclusion

Our data suggest that diet-induced obesity results in increased infiltration of peripheral myeloid cells into the brain. Cells with similar gene expression signatures appear in the brains of aged mice and a mouse model of AD. These cells are likely composed of monocytes and neutrophils and may elicit proinflammatory responses  that destabilizes the blood-brain barrier and/or brain function. Understanding their contribution to diet-induced obesity will allow us to determine neuroinflammatory components that are shared with age-related cognitive decline and other age-related neurodegenerative diseases such as Alzheimer’s disease, where diet/obesity are major risk factors.

## Additional files

Additional file 1: **Figure S1.** The percent of CD45+CD11c+ cells was increased by WD consumption in a CCR2-dependent manner. **(A)** Gating strategy showing total CD45+CD11c+ cells from total CD45+CD11b+ cells (from Fig. 3f). **(B-C)** Gating strategy showing CD11c+ cells from CD11b+CD45^lo^ (B) and CD11b+CD45^hi^ cells (C) from Fig. 3g, respectively. (D) Box plot showing the percent of total CD45+CD11c+ cells from 6-month CD-fed, 12-month CD- or WD-fed WT or *Ccr2*-KO mice. (E) Box plot showing the percent of CD45^lo^CD11c+ (marked in B) and CD45^hi^CD11c+ (marked in C) cells (two-way ANOVA followed by Tukey HSD post hoc test, *p < 0.05, **p < 0.01, ***p < 0.001). **Figure S2.** Ly6c+Ly6g− and Ly6c+Ly6g+ cells are predominantly expressed in CD11b+CD45^hi^ cells. **(A)** Gating strategy showing total Ly6c+Ly6g− and Ly6c+Ly6g+ cells from total CD45+CD11b+ cells (from Fig. 3f). **(B-C)** Gating strategy showing Ly6c+Ly6g− and Ly6c+Ly6g+ cells were profiled from CD11b+CD45^lo^ (B) and CD11b+CD45^hi^ cells (C) from Fig. 3g, respectively. **(D)** Box plot showing the percent of total Ly6c+Ly6g− cells from 6-month CD-fed, 12-month CD or WD-fed WT or *Ccr2*-KO mice. **(E)** Box plot showing the percent of CD45^lo^Ly6c+Ly6g− (B) and CD45^hi^Ly6c+Ly6g− (C) cells. **(F)** Box plot showing the percent of total Ly6c+Ly6g+ cells in the same groups of mice. **(G)** Box plot showing the percent of CD45^lo^Ly6c+Ly6g+ (B) and CD45^hi^Ly6c+Ly6g+ (C) cells (two-way ANOVA followed by Tukey HSD post hoc test, ***p < 0.001). **Figure S3.** The percent of T cells was increased during aging in a Ccr2-dependent manner. Box plots showing the percent of CD45+CD3e+ cells from 6-month CD-fed, 12-month CD or WD-fed WT or *Ccr2*-KO mice. The gating strategy was shown in Fig. 3f (Two-way ANOVA followed by Tukey HSD post hoc test, ***p < 0.001). **Figure S4.** Gene expression of major cell type markers in the brain. Box plots showing expression levels of marker genes of myeloid cells, astrocytes, pericytes, endothelial cells, and oligodendrocytes in CD11b+C45^lo^ and CD11b+CD45^hi^ cells from CD or WD-fed mice. **Figure S5.** WD may affect protein ubiquitination pathway and *Ctsd* expression in CD11b+CD45^hi^ cells. (A) Canonical pathways by IPA enriched in DE genes comparing CD11b+CD45^hi^ cells between WD and CD-fed mice, [−log(*p* value)] > 1.3 and number of genes in pathways ≥ 3. (B) Box plots showing the log_2_-transformed expression of *Ctsd* and genes involved in protein ubiquitination pathway in CD11b+C45^lo^ and CD11b+CD45^hi^ cells from CD or WD-fed mice. (C) Fold change (FC) of *Ctsd* and genes involved in protein ubiquitination pathway comparing CD11b+CD45^hi^ cells from WD and CD-fed mice, colored by significance level using -log_10_(FDR). The positive FC value means the gene expression of CD11b+CD45^hi^ from WD is larger in than that in CD-fed mice and vice versa. **Figure S6.** Shared transcriptomic features of brain myeloid cells in diet-fed, aged B6 and B6.*APP/PS1* mice. **(A)** PCA plot showing the first and second component of transcriptional expression profiles between CD11b+C45^lo^ and CD11b+CD45^hi^ cells in aged WT (20 months) mice and APP/PS1 (6 months) mice. **(B)** Top 15 shared canonical pathways revealed by IPA based on DE genes between CD11b+C45^lo^ and CD11b+CD45^hi^ cells in mice fed a CD or WD (12 months), aged WT mice (20 months) and APP/PS1 (6 months). **Figure S7.** Top genes enriched in CD11b+CD45^hi^ cells reflected peripheral myeloid cell profiles in WD-fed mice. Normalized gene expression plot **(**reproduced from ImmGen datasets) showing relative gene expression values for 34 CD11b+C45^lo^ cell-enriched DE genes (A) or 73 CD11b+CD45^hi^ cell-enriched DE genes (B) across all immune cell types available on ImmGen RNA-seq datasets. **Figure S8.** The number of OPN+IBA1+ cells per animal in the brain. (**A**) Box plot showing the number of OPN+IBA1+ cells per animal (the sum of cell numbers on seven images) in 12-month CD or WD-fed WT mice. (*T* test, **p = 0.0021). (**B**) Box plot showing the number of OPN+IBA1+ cells per animal (the sum of cell numbers on seven images) in 12-month CD or WD-fed *Ccr2*-KO mice. (*T* test; not significant, NS). (PDF 7836 kb)

Additional file 2: Gene list comparing the transcriptomes of CD11b+CD45^lo^ with CD11b+CD45^hi^ cells in WD-fed mice. Pairwise comparison of transcriptomes between CD11b+CD45^lo^ and CD11b+CD45^hi^ cells in WD-fed mice. The positive FC value means the gene expression is larger in CD11b+CD45^hi^ than in CD11b+CD45^lo^ and vice versa. DE genes were defined as FDR < 0.05. (XLSX 1720 kb)

Additional file 3: Gene list comparing the transcriptomes of CD11b+CD45^lo^ with CD11b+CD45^hi^ cells in CD-fed mice. Pairwise comparison of transcriptomes between CD11b+CD45^lo^ and CD11b+CD45^hi^ cells in CD-fed mice. The positive FC value means the gene expression is larger in CD11b+CD45^hi^ than in CD11b+CD45^lo^ and vice versa. (XLSX 1700 kb)

Additional file 4: Gene list comparing the transcriptomes of CD11b+CD45^hi^ from CD-fed mice and WD-fed mice. The positive FC value means the gene expression CD11b+CD45^hi^ is larger in WD-fed mice than that in CD-fed mice and vice versa. DE genes were defined as FDR < 0.05. (XLSX 1730 kb)

Additional file 5: The top CD11b+CD45^lo^ cell-related genes in WD-fed mice. The top DE genes enriched in CD11b+CD45^hi^ cells were defined as those with expression levels above 100 cpm and at least two-fold higher compared to CD11b+CD45^lo^ cells. (XLSX 14 kb)

Additional file 6: The top CD11b+CD45^hi^ cell-related genes in WD-fed mice. The top DE genes enriched in CD11b+CD45^hi^ cells were defined as those with expression levels above 100 cpm and at least 10-fold higher compared to CD11b+CD45^lo^ cells. (XLSX 20 kb)

## Data Availability

All raw fastq files and processed gene expression read counts for each animal can be found in NIH GEO Archive (GSE133814).

## Abbreviations

AD: Alzheimer's disease
B6: C57BL/6J
BBB: Blood-brain barrier
BJ: Benjamini *p* value
BP: Biological process
CC: Cellular component
CD: Control chow diet
cpm: Counts per million
DAM: Disease-associated microglia
DE: Differentially expressed
FACS: Fluorescent activated cell sorting
FDR: False discovery rate
GO: Gene ontology
ImmGen: Immunological Genome Project
IPA: Ingenuity Pathway Analysis
KO: Knockout
OPN: Osteopontin
PCA: Principal component analysis
WD: Western diet
