# Supplementary figures and images for: Transcriptome profiling of brain myeloid cells revealed activation of Itgal, Trem1, and Spp1 in western diet-induced obesity
Source: J Neuroinflammation. 2019 Aug 19;16:169. doi: 10.1186/s12974-019-1527-z (PMC6700800; doi:10.1186/s12974-019-1527-z)

Fig S1

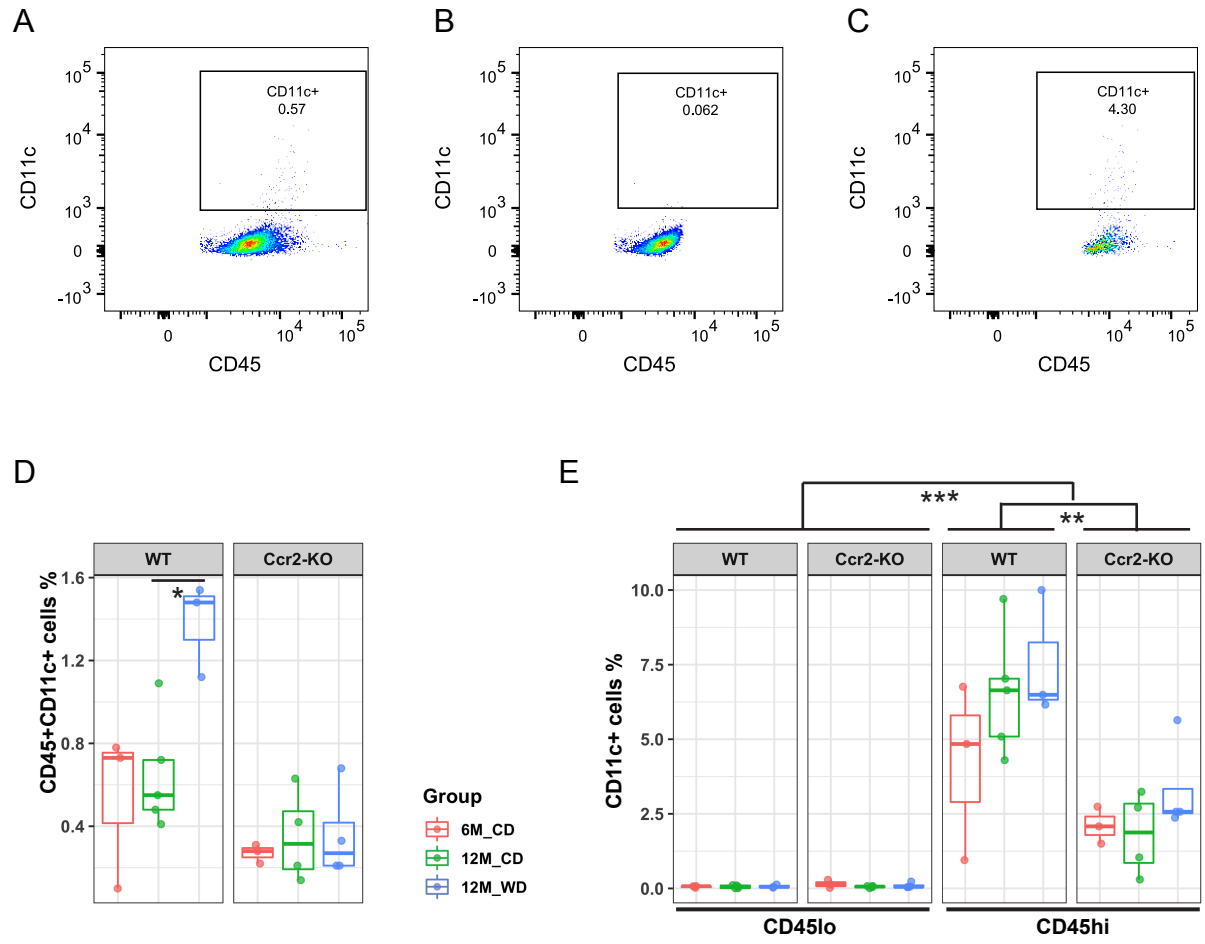

Fig. S2

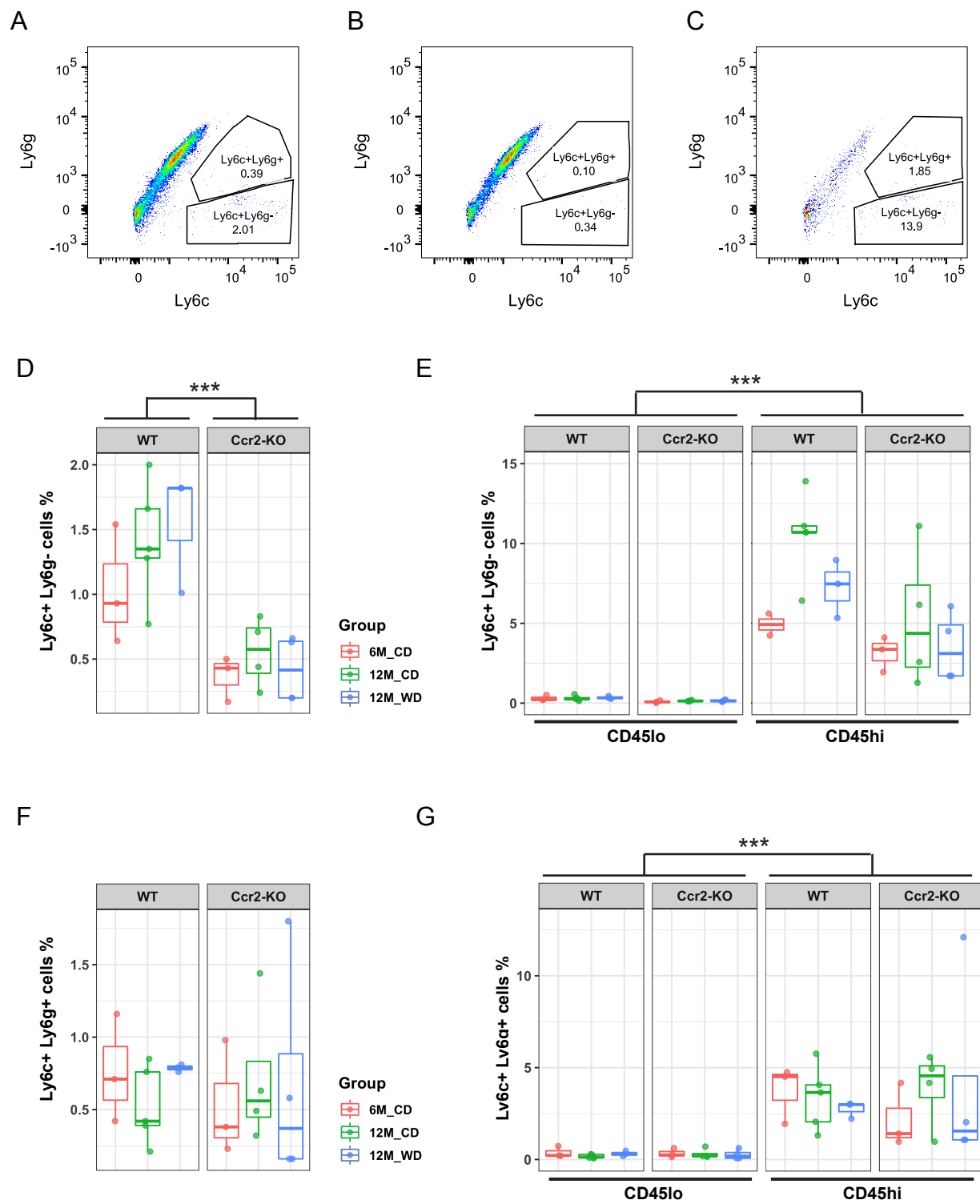

Fig S3

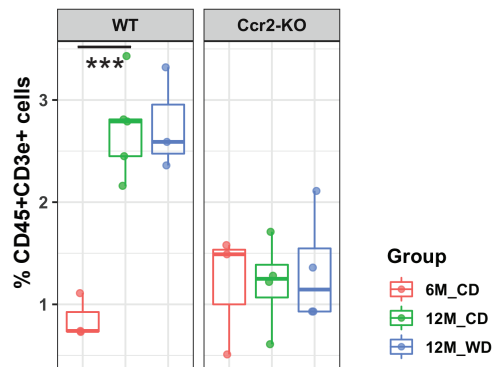

Fig S4

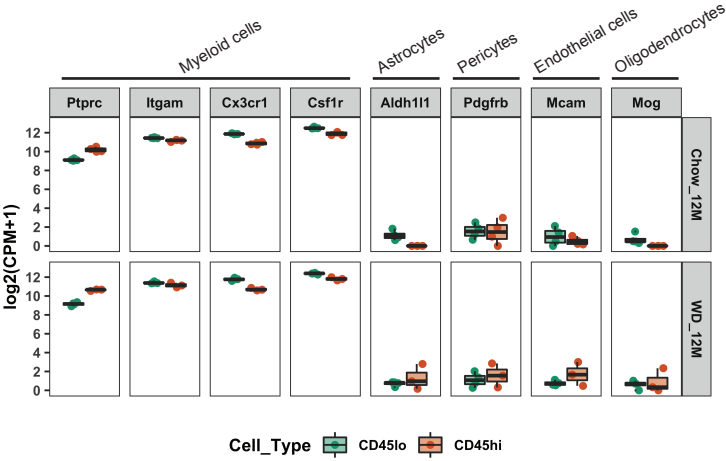

Fig S5

A

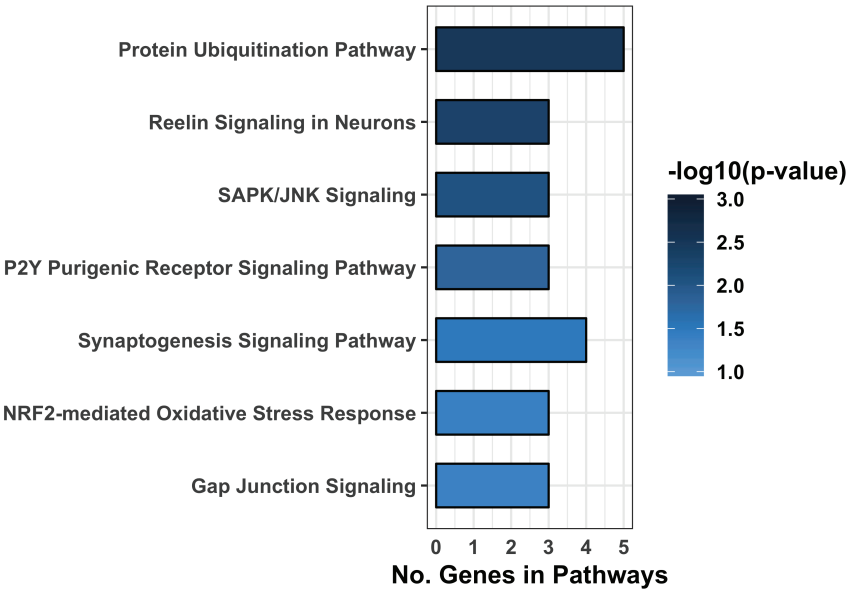

B

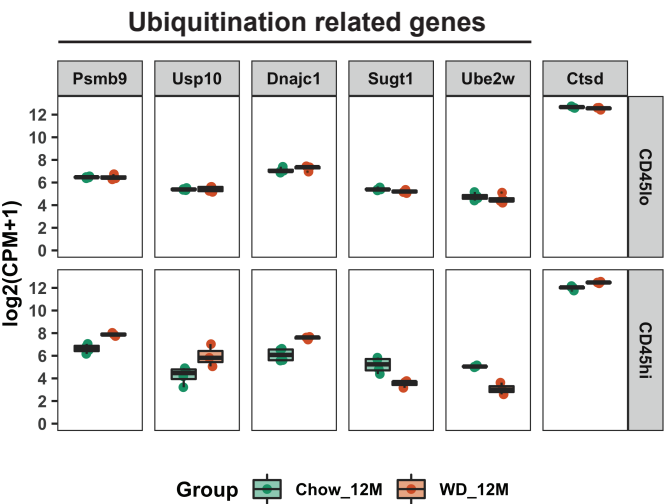

C

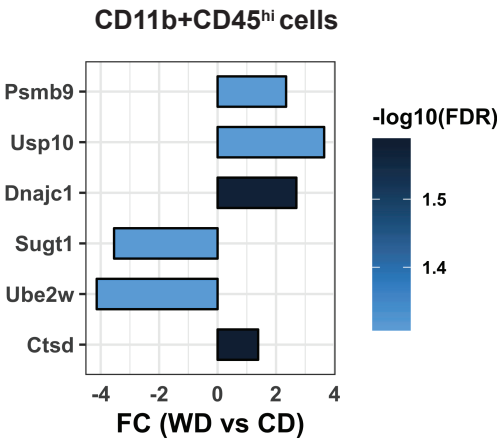

Fig S6

A

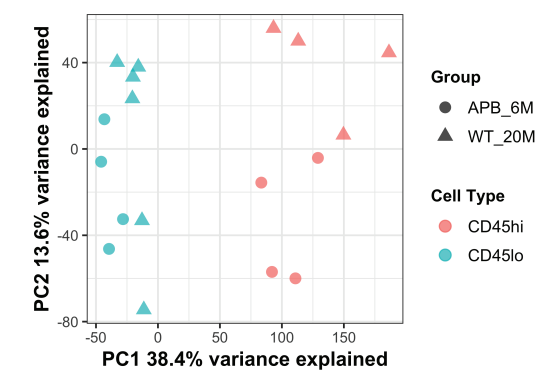

B

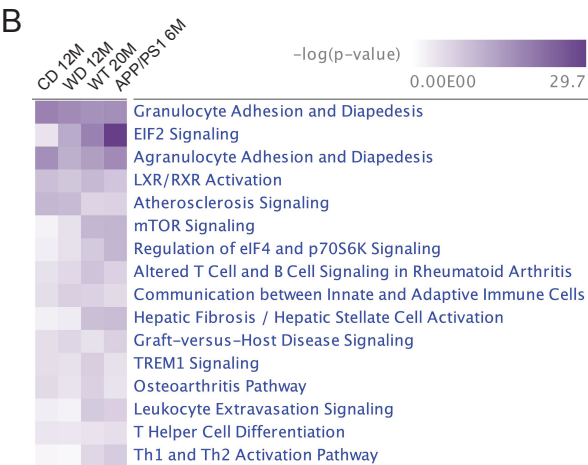

Fig S7

A

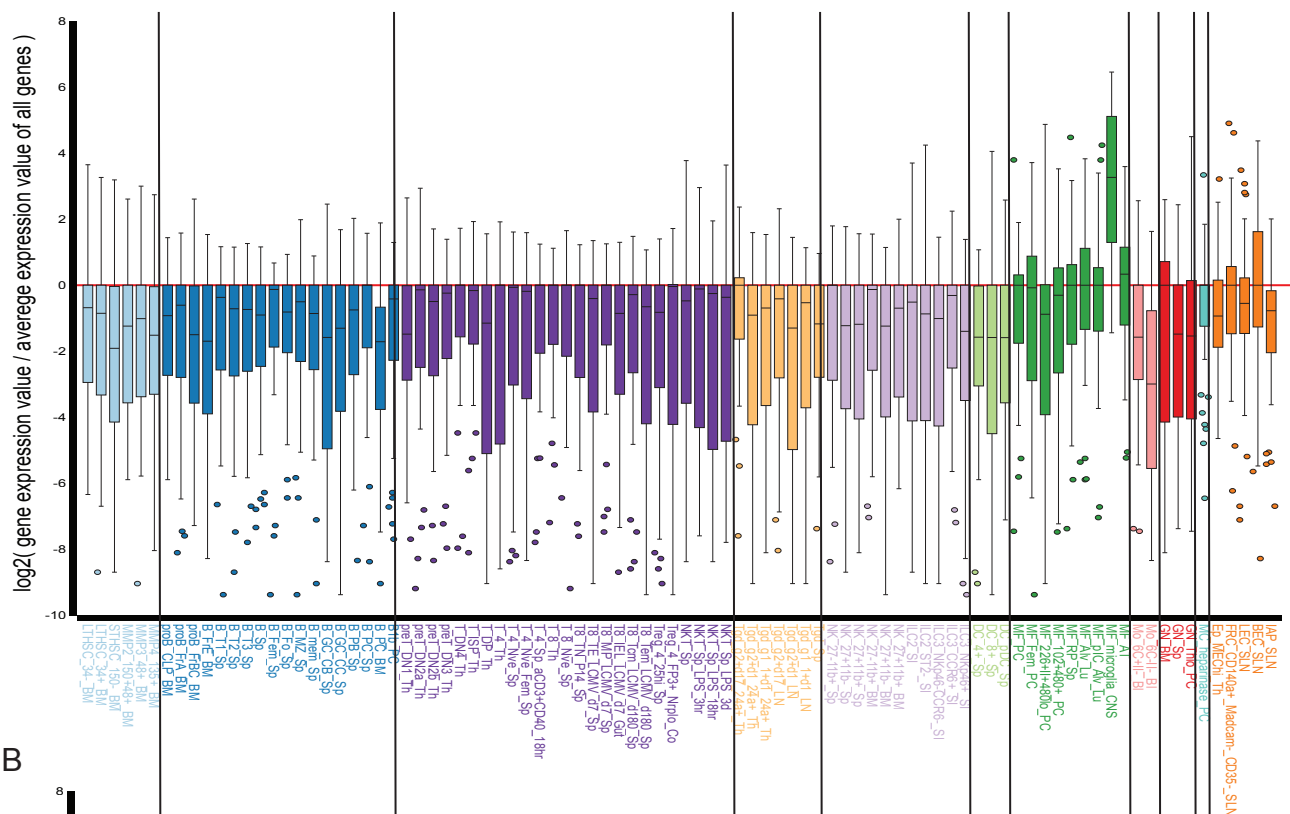

B

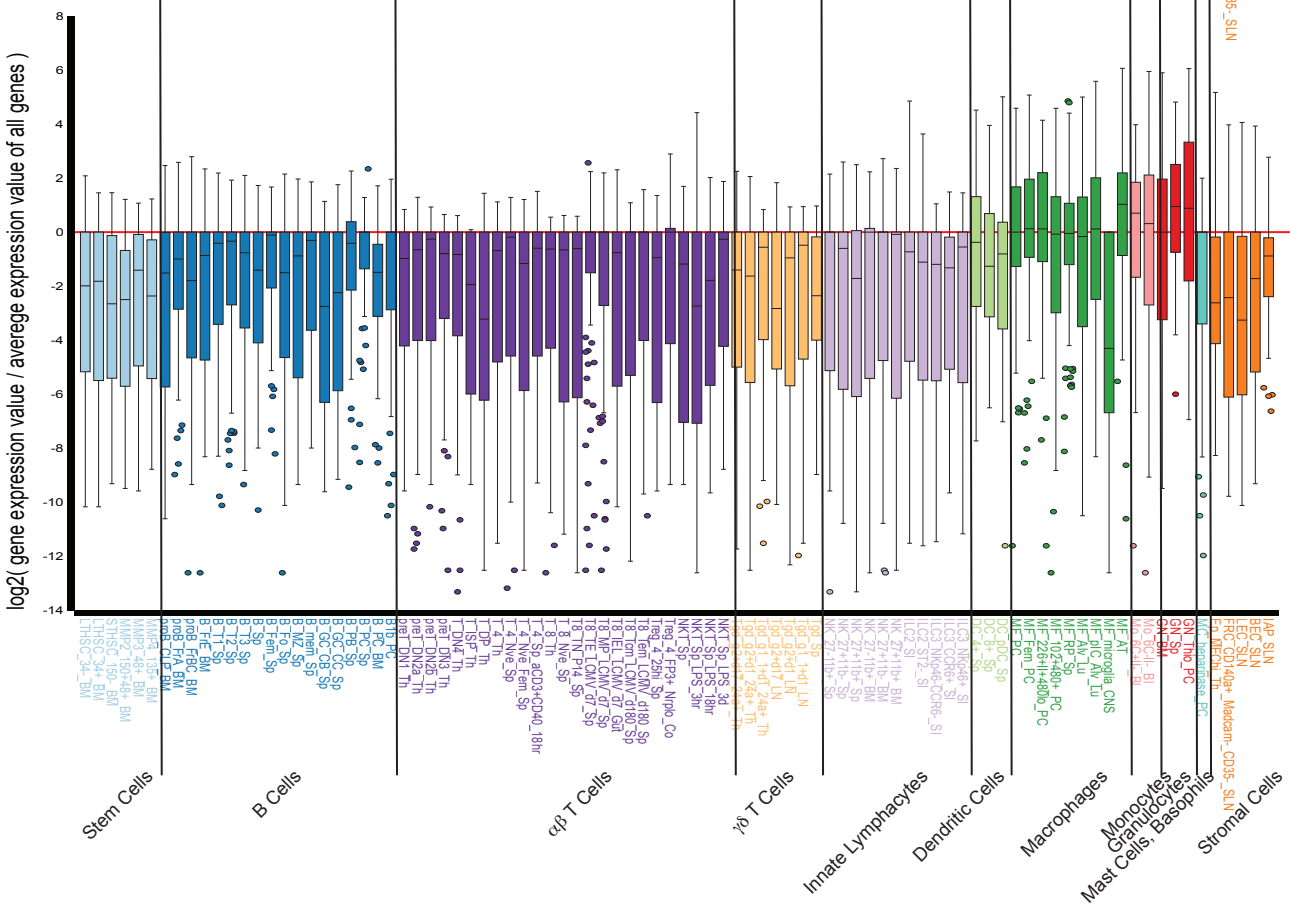

Fig S8

A

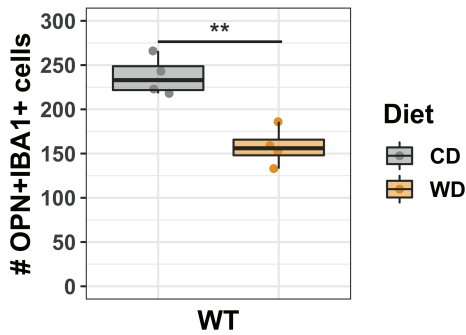

B

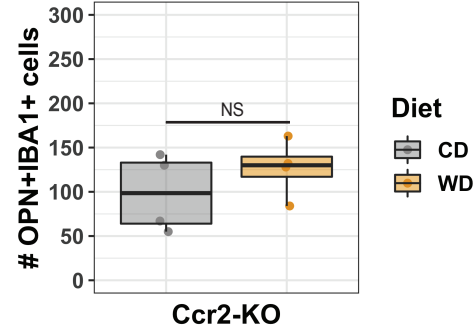

Supplement: Supplementary file 1 — Figure S1. The percent of CD45+CD11c+ cells was increased by WD consumption in a CCR2-dependent manner. (A) Gating strategy showing total CD45+CD11c+ cells from total CD45+CD11b+ cells (from Fig. 3f). (B-C) Gating strategy showing CD11c+ cells from CD11b+CD45lo (B) and CD11b+CD45hi cells (C) from Fig. 3g, respectively. (D) Box plot showing the percent of total CD45+CD11c+ cells from 6-month CD-fed, 12-month CD- or WD-fed WT or Ccr2-KO mice. (E) Box plot showing the percent of CD45loCD11c+ (marked in B) and CD45hiCD11c+ (marked in C) cells (two-way ANOVA followed by Tukey HSD post hoc test, *p < 0.05, **p < 0.01, ***p < 0.001). Figure S2. Ly6c+Ly6g− and Ly6c+Ly6g+ cells are predominantly expressed in CD11b+CD45hi cells. (A) Gating strategy showing total Ly6c+Ly6g− and Ly6c+Ly6g+ cells from total CD45+CD11b+ cells (from Fig. 3f). (B-C) Gating strategy showing Ly6c+Ly6g− and Ly6c+Ly6g+ cells were profiled from CD11b+CD45lo (B) and CD11b+CD45hi cells (C) from Fig. 3g, respectively. (D) Box plot showing the percent of total Ly6c+Ly6g− cells from 6-month CD-fed, 12-month CD or WD-fed WT or Ccr2-KO mice. (E) Box plot showing the percent of CD45loLy6c+Ly6g− (B) and CD45hiLy6c+Ly6g− (C) cells. (F) Box plot showing the percent of total Ly6c+Ly6g+ cells in the same groups of mice. (G) Box plot showing the percent of CD45loLy6c+Ly6g+ (B) and CD45hiLy6c+Ly6g+ (C) cells (two-way ANOVA followed by Tukey HSD post hoc test, ***p < 0.001). Figure S3. The percent of T cells was increased during aging in a Ccr2-dependent manner. Box plots showing the percent of CD45+CD3e+ cells from 6-month CD-fed, 12-month CD or WD-fed WT or Ccr2-KO mice. The gating strategy was shown in Fig. 3f (Two-way ANOVA followed by Tukey HSD post hoc test, ***p < 0.001). Figure S4. Gene expression of major cell type markers in the brain. Box plots showing expression levels of marker genes of myeloid cells, astrocytes, pericytes, endothelial cells, and oligodendrocytes in CD11b+C45lo and CD11b+CD45hi cells from [file 12974_2019_1527_MOESM1_ESM.pdf]
